# Use of Nanoparticles As Contrast Agents for the Functional and Molecular Imaging of Abdominal Aortic Aneurysm

**DOI:** 10.3389/fcvm.2017.00016

**Published:** 2017-03-23

**Authors:** Theophilus I. Emeto, Faith O. Alele, Amy M. Smith, Felicity M. Smith, Tammy Dougan, Jonathan Golledge

**Affiliations:** ^1^Public Health and Tropical Medicine, College of Public Health, Medical and Veterinary Sciences, James Cook University, Townsville, QLD, Australia; ^2^Queensland Research Centre for Peripheral Vascular Diseases, College of Medicine and Dentistry, James Cook University, Townsville, QLD, Australia; ^3^Department of Medicine, University of Cambridge, Cambridge Biomedical Campus, Addenbrookes Hospital, Cambridge, UK; ^4^Department of Vascular and Endovascular Surgery, The Townsville Hospital, Townsville, QLD, Australia

**Keywords:** abdominal aortic aneurysm, nanoparticles, diagnosis, human studies, animal studies

## Abstract

Abdominal aortic aneurysm (AAA) is a degenerative disease of the aorta common in adults older than 65 years of age. AAA is usually imaged using ultrasound or computed tomography. Molecular imaging technologies employing nanoparticles (NPs) have been proposed as novel ways to quantify pathological processes, such as inflammation, within AAAs as a means to identify the risk of rapid progression or rupture. This article reviews the current evidence supporting the role of NP-based imaging in the management of AAA. Currently, ultrasmall superparamagnetic NPs enhanced magnetic resonance imaging appears to hold the greatest potential for imaging macrophage-mediated inflammation in human AAA.

## Introduction

Abdominal aortic aneurysm (AAA) is a degenerative disease of the aorta common in older adults (1–4). AAA is usually defined as a macroscopic permanent pathological dilatation of the infrarenal aortic diameter ≥30 mm (4–7). Most AAAs are asymptomatic until rupture, which is often fatal (1, 3, 4, 8). Patients with small AAAs usually undergo regular imaging to monitor AAA diameter until it exceeds 55 mm (4). At this point, patients are usually recommended to have open surgical or endovascular stent graft repair according to current guidelines (2, 8, 9). AAA management is primarily focused on averting rupture (8, 9). Rupture of small AAAs is uncommon (2, 10). It has been reported that approximately 1% of AAAs measuring <55 mm rupture each year while undergoing careful follow-up during which large, symptomatic, or progressively expanding AAAs are selected for surgical repair (11–13). For example, the UK small aneurysm trial reported that the rupture rate for asymptomatic AAAs measuring <55 mm was 1% per annum, and the risk of rupture was higher in women (12). The future management of AAA could be optimized through an enhanced ability to identify small AAAs at risk of rupture and identification of small AAAs that would benefit from early surgical intervention (3, 14). One potential way of doing this could be through functional or molecular imaging in which key pathological processes implicated in AAA rupture were quantified in individual patients.

**Figure 1:**
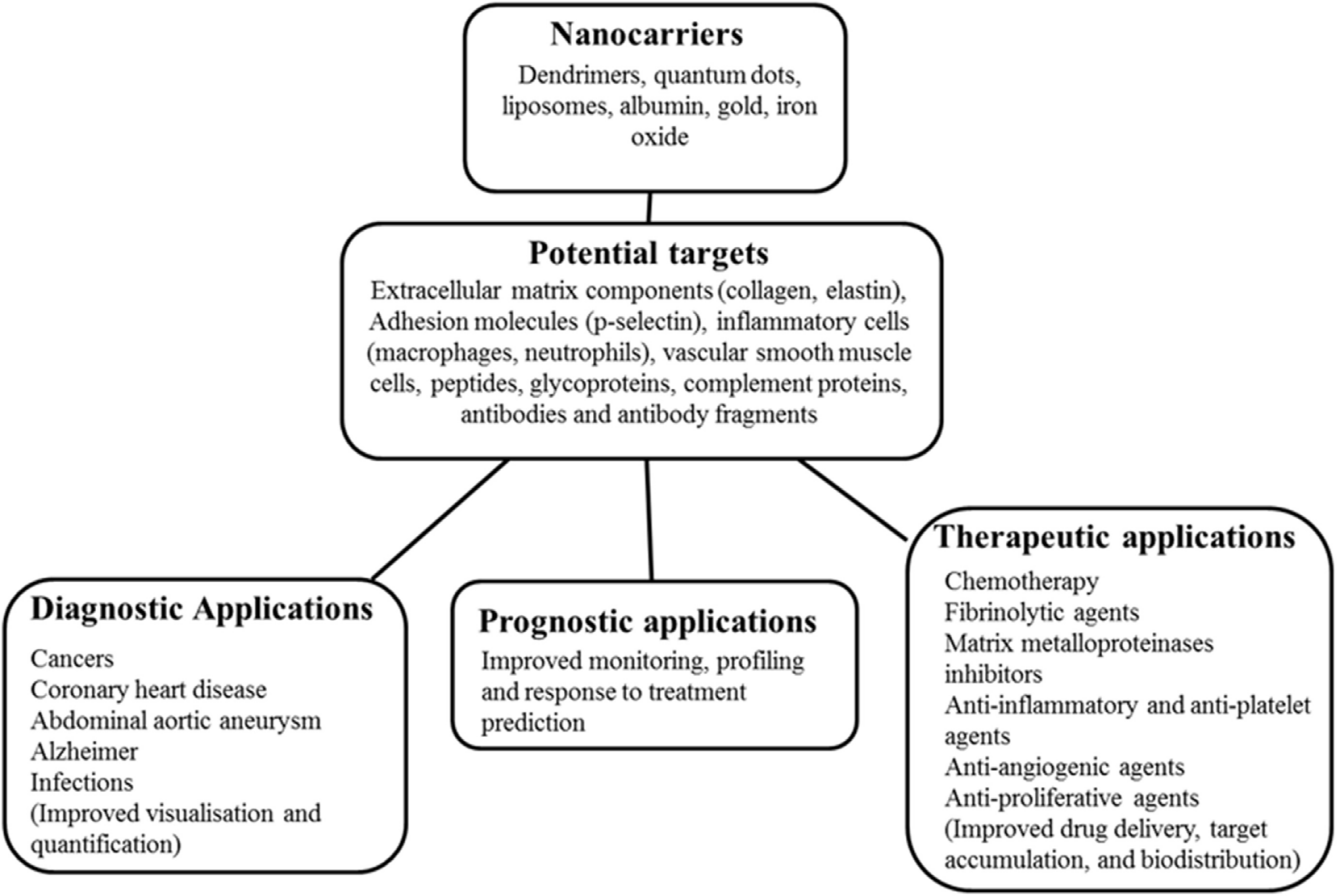
**Schematic diagram of nanocarriers, potential targets, and potential applications in medicine**. This shows nanocarriers with typical nanoparticle (NP) base/core, potential targets in humans ranging from receptors, immune fragments, and vascular tissue components. The potential role of the NPs in disease management in medicine is also highlighted.

Recent research has explored the efficacy of nanoparticle (NP)-enhanced molecular imaging technologies in identifying key pathological processes within AAAs (15, 16). NPs are constructs of sizes ranging from 1 to 100 nm in at least one dimension and are 100 to 10,000 times smaller than human cells (17–19). They are bioactive and can interact with biological molecules both intra and extracellularly (20). Generally, they are characterized by a long blood half-life and can evade elimination by the reticuloendothelial system (17, 21); hence, they are relatively stable in biological systems. NPs can be successfully delivered using optimized nanocarriers such as dendrimers, quantum dots, liposomes, albumin, gold, and iron oxide to target diseased or normal tissues as therapeutic or diagnostic agents (Figure 1) (19, 21, 22). Their unique properties include a high penetration power (23), ability to be modified with any molecule of choice (24–26), biocompatible size distribution (27), and image contrasting ability (28). The use of NPs in medicine (nanomedicine) is being investigated in a range of diseases, particularly cancers (17) and cardiovascular disease (29–31). Nanotube-antibody microarrays, for example, have been used to detect metastatic breast cancer cells in the circulation (32). NPs have been employed in imaging AAA (33, 34) and to enhance magnetic resonance imaging (MRI) detection of endoleaks following endovascular aneurysm repair (29). NP-based biofilms have been utilized to detect infection (35).

Magnetic NPs and quantum dots are being investigated as treatments to modify stem cell proliferation and differentiation in regenerative medicine (36, 37). For example, mice with spinal cord injury injected with nanofiber conjugated with laminin were reported to show improved neurological function (36). Polylactic-*co*-glycolic acid NPs conjugated to tissue plasminogen activator have been reported to gradually lyse fibrin-rich clots (38). This has been suggested as a potential strategy for removing intraluminal thrombus (ILT) to allow endovascular delivery of therapeutic agents to the wall of AAAs, although this has not been specifically investigated. Intra-arterial NP-based thrombolytic therapy combined with temporary endovascular bypass has also been reported to be more effective than temporary endovascular bypass alone in a rabbit model of carotid artery occlusion (39). This may potentially be important in the management of patients with arterial occlusion. For other potential applications of NPs in medicine, please refer to the articles by Wang and Wang (40) and Zhang et al. (41). A number of animal and human investigations have studied various NPs as nanocarriers or in combination with standard contrast agents in imaging AAA (33, 34, 42–45).

In this article, we discuss the findings of these studies and highlight the potential challenges in utilizing these novel molecules as contrast agents in the functional and molecular imaging of AAA.

## Literature Search

A literature search was conducted to identify studies employing NPs for molecular imaging of AAA using the MEDLINE (1966), SCOPUS (1996), Web of Science (1965), and Cochrane Library databases (1992) from inception to the 25th of September 2016. The following search terms were applied either as single or combined searches: “abdominal aortic aneurysm diagnosis” OR “AAA imaging,” [Title/Abstract] AND “nanoparticles,” AND/OR “clinical studies” OR “human studies,” AND/OR “animal studies” OR “experimental studies.” Abstracts were analyzed for relevance. Studies describing the use of NPs as agents for AAA imaging were retrieved. All studies investigating the use of NPs in AAA imaging were included. The reference lists of all included articles were also hand searched. Studies excluded were those in languages other than English and investigations which did not use NPs in AAA imaging.

## AAA Pathogenesis and Potential Imaging Targets for NPs

**Figure 2:**
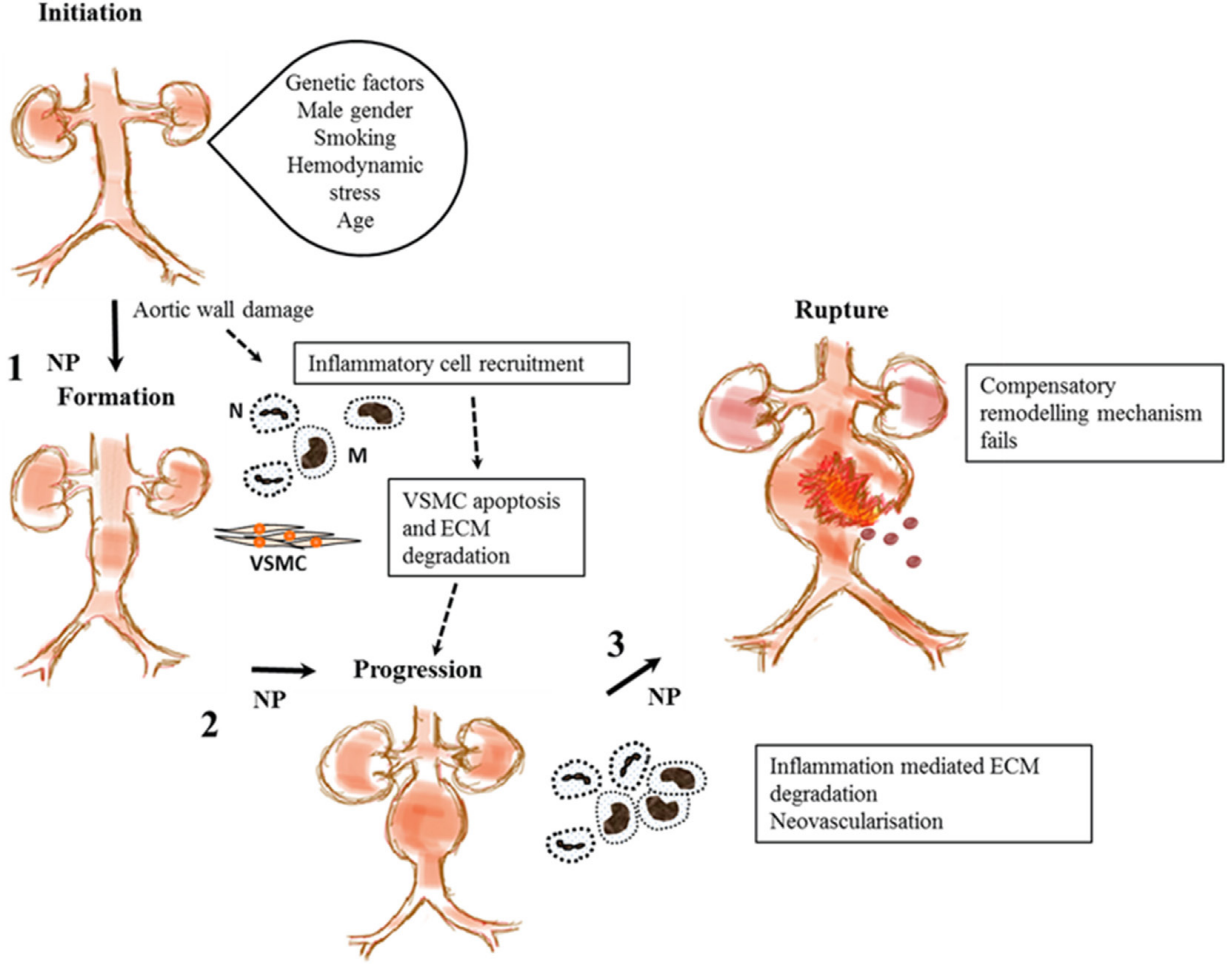
**Proposed stages in abdominal aortic aneurysm (AAA) pathogenesis and steps where nanoparticles (NPs) have been used to enhance imaging the disease process**. This shows proposed stages in AAA pathogenesis from initiation to rupture. A combination of genetic and environment factors and tissue injury may lead to recruitment of inflammatory cells, macrophages (M), and neutrophils (N) leading to vascular smooth muscle cell (VSMC) apoptosis and extracellular matrix (ECM) degradation. This leads to aortic wall weakening and subsequent aneurysm progression. The aorta tries to repair itself but is overwhelmed by continual inflammation and ECM degradation. Eventually, failure of this compensatory mechanism leads to aortic rupture and death. NPs target inflammatory cells and components of the ECM at steps 1, 2, and 3 to aid in the imaging of functional components implicated in AAA pathology.

Abdominal aortic aneurysm is a complex disease thought to be an abnormal interaction between genetic predisposition and environmental risk factors that aggravate the normal aging processes (3). It has been suggested that AAA formation is initiated by endothelial injury with resultant chronic inflammation denoted by invasion of the *tunica media* by inflammatory cells including lymphocytes and macrophages as shown in Figure 2 (46–51). Macrophages in turn secrete proteolytic enzymes such as matrix metalloproteinases (MMPs) (52, 53), resulting in significant remodeling and degradation of the extracellular matrix (ECM), significant damage to elastin and collagenous fibers and reductions in vascular smooth muscle cell (VSMC) density (53, 54). We and others have shown that most AAAs have marked ILT that is implicated in AAA progression (4, 55). Previous research suggests that ILT encourages the migration of neutrophils (56), macrophages, and lymphocytes (48), which are implicated in VSMC apoptosis and degradation of the aortic wall (48). P-selectin, an adhesion molecule expressed by the endothelium and activated platelet, mediates leukocyte diapedesis and trapping (57, 58) and is implicated in ILT growth that may be important in AAA progression (4, 57).

Macrophage- and lymphocyte-driven inflammation is believed to be a key factor in AAA pathogenesis (2, 4, 6, 7). This is evidenced by previous research from our group and others demonstrating marked infiltration of macrophages, lymphocytes, and neutrophils in aneurysmal tissue (1, 46). Iron oxide NPs have been reported to have a high affinity for macrophages (48–50). Hence, these NPs, specifically the subset with diameters <50 nm known as ultrasmall superparamagnetic iron oxide nanoparticles (USPIOs), have been used as agents to aid imaging of AAAs in both animal models and patients (30–35). USPIOs such as ferumoxytol are composed of an iron oxide core enclosed by a hydrophilic coating that readily accrues in neutrophils and macrophages (51). They have been employed as MRI contrasts agents for assessing tissues with active inflammation such as AAA. NPs have been employed as agents to target inflammation (macrophages, neutrophils), VSMC apoptosis, and ECM degradation (P-selectin) in AAA.

## Studies Assessing NPs in AAA Imaging

Several molecular imaging approaches have been investigated for AAA, but their ability to clearly differentiate between AAAs at risk of rupture and predict AAAs that will benefit from a surgical intervention is still unclear (3, 14, 15, 59). Both animal and human studies have suggested that NPs, particularly those with an iron oxide component, can be taken up by AAAs and identified on imaging (30–35). We highlight some of these studies in the following sections.

### Animal Studies

**Figure 3:**
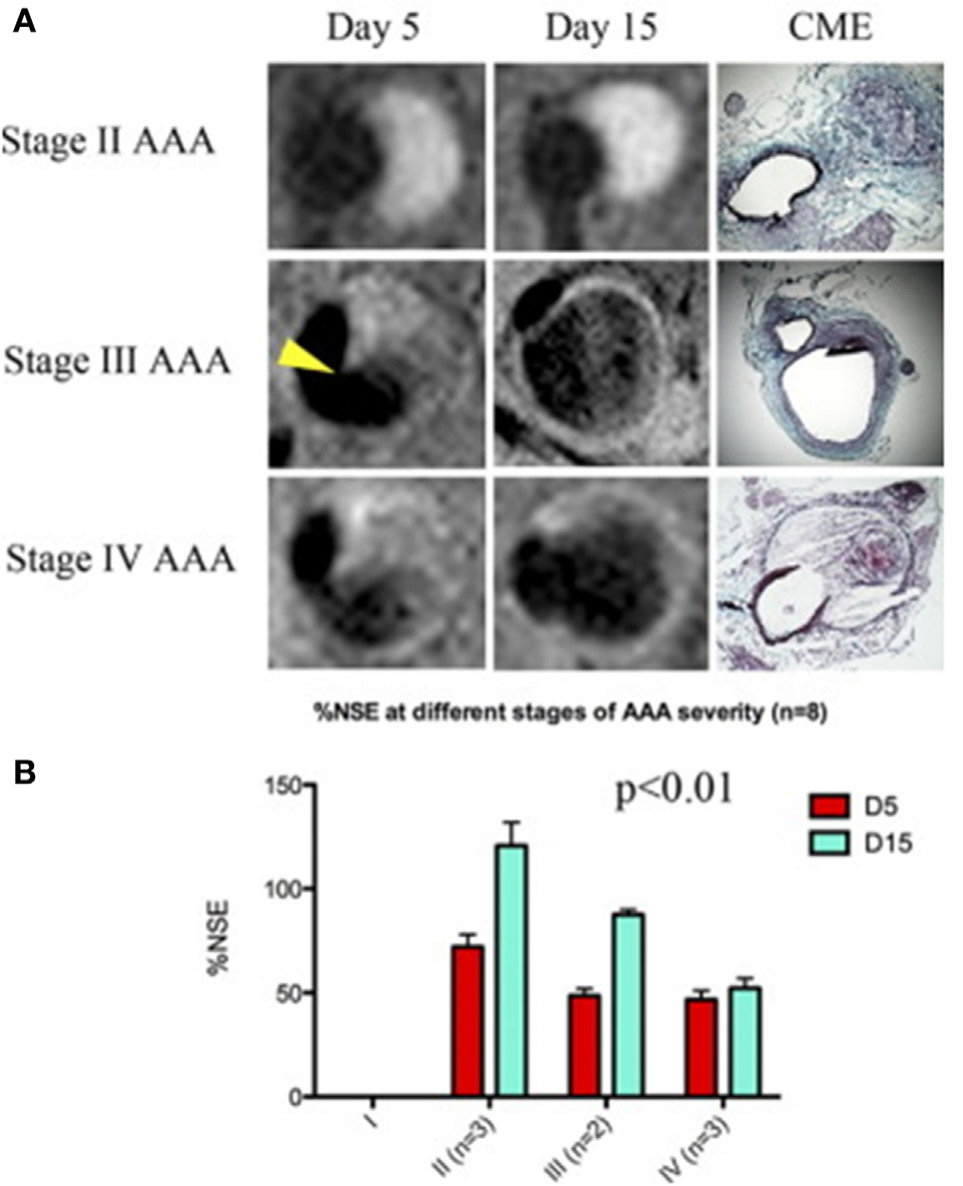
**CNA-35-enhanced MR imaging of AAAs of increasing severity at early and late stage of development and complications**. **(A)** Typical image of Stages II–IV AAA obtained after CNA-35 injection. Corresponding histological sections stained with combined Masson elastin are shown in the third column. **(B)** Quantification of aneurysm severity (increasing from Stage I–IV) and normalized signal enhancement percentage (%NSE) relative to CAN-35 injection. Reprinted from Klink et al. (31), with permission from Elsevier. Abbreviations: %NSE, normalized percentage signal enhancement; AAA, abdominal aortic aneurysm; CNA-35, paramagnetic/fluorescent micellar nanoparticles functionalized with a collagen-binding protein. AAA severity Stages I–IV are based on the classification by Manning et al. (64).

A number of studies have assessed the use of NPs to enhance imaging of AAA within animal models (Table 1) (58, 60, 61). ILT is common within AAAs, and a large volume of thrombus has been associated with more rapid AAA progression (62). Suzuki and colleagues reported that the detection of ILT within a rat model using MRI was enhanced by infusion of USPIOs coated with fucoidan (USPIO-FUCO) as compared with infusion of USPIOs coated with carboxymethyldextran (USPIO-CMD) (61). Fucoidan is a natural ligand for P-selectin with high affinity for activated platelets (61). The authors reported that intraluminal hyposignals detected by USPIO-FUCO enhanced MRI where histologically confirmed to be thrombus. Bonnard et al. using a rat model reported that fucoidan-conjugated microparticle-enhanced MRI detection of inflammatory cells localized in AAAs (58). Turner and colleagues evaluated the use of the USPIO (ferumoxtran) as a marker for the detection of macrophages in the angiotensin II (ang II)-infused apolipoprotein E deficient (ApoE^−/−^) AAA mouse model (45). They reported marked accumulation of USPIO-labeled macrophages within the aneurysmal aorta that could be identified by MRI and was confirmed by immunohistochemistry (45). Similarly, Yao et al. reported that superparamagnetic iron oxide (SPIO) enhanced MRI visualization of AAA in an angiotensin II-infused ApoE^−/−^ model of AAA (42). They proposed that SPIO diffuses across the interendothelial junction of the *vasa vasorum* into the interstitium where they are engulfed by macrophages. These macrophages then migrate into the *tunica media* and *tunica adventitia* within the aneurysmal aorta. The authors suggested that SPIO-enhanced imaging may be useful for quantifying the risk of AAA rupture (42). This assertion by the authors is mitigated by the fact that the presence of endogenous iron within ILT may reduce the specificity of SPIO-enhanced imaging for localizing inflammation. ILT is reported to be rich in inflammatory cells (55, 63). In a mouse model in which AAAs were induced by ang II infusion and injection of transforming growth factor-β neutralizing antibody, Klink and colleagues reported that administration of paramagnetic/fluorescent micellar NPs functionalized with a collagen-binding protein (CNA-35) markedly increased high-resolution multi-sequence MRI visualization of aortic remodeling (31). AAA severity (Stages I–IV) was based on the classification by Alan Daugherty’s group (64). They imaged mice in which AAAs had been induced 5 and 15 days after CNA-35 NP injections and correlated the images to disease pathology (Figure 3). Marked CNA-35 NP uptake correlated with high collagen uptake (Stage II AAA) and hence less ECM degradation, while rupture Stage IV AAAs had negligible CNA-35 NP and low collagen uptake. These data suggested that CNA-35 micelles were able to identify some AAA pathological changes, but how this relates to rupture risk in patients is unknown (31).

**Table 1:** **Examples of animal studies assessing the use of nanoparticles (NPs) in AAA imaging**.

AAA model	AAA process	Target	NP	Imaging mode	Findings
Elastase-induced rat model	Inflammation	Platelets expressing P-selectin	USPIO-FUCO	MRI	USPIO-FUCO-enhanced MRI detection of ILT (61)
Ang II-infused Apo E^−/−^ mice	Inflammation	Macrophages	USPIO	MRI	Reduced signal intensity in the post-USPIO transverse images of AAA (45)
Ang II-infused Apo E^−/−^ mice	Inflammation	Macrophages	SPIO	MRI	Ang II infusion increased SPIO uptake. AAA wall contained significantly more iron-positive macrophages (42)
Ang II-infused and TGβ-neutralized C57BL/6 mice	ECM remodeling	Collagen	CNA-35 micelles	MRI	Increased MRI signal enhancement in AAA wall (31)
Ang II-infused Apo E^−/−^ mice	Inflammation	Macrophages	RGD-HFn	NIR and MRI	RGD-HFn-enhanced NIR (65), and MRI (30) imaging of AAA
	Angiogenesis	Endothelial cells			
Ang II-infused Apo E^−/−^ mice	Inflammation	Macrophages	^18^F-CLIO	PET-CT	Improved PET-CT imaging of AAA (66)

*^18^F-CLIO, dextran-coated cross linked-iron oxide nanoparticles labeled with fluorine-18; AAA, abdominal aortic aneurysm; Ang II, angiotensin II; ApoE^−/−^, apolipoprotein E deficient; C57BL/6, C57 black 6; CNA-35, paramagnetic/fluorescent micellar nanoparticles functionalized with a collagen-binding protein; ECM, extracellular matrix; ILT, intraluminal thrombus; MRI, magnetic resonance imaging; NIR, near-infrared fluorescence imaging; PET-CT, positron emission tomography-computed tomography; RGD-HFn, Arg–Gly–Asp-conjugated human ferritin nanoparticle; SPIO, superparamagnetic iron oxide nanoparticles; TGFβ, transforming growth factor-β; USPIO, ultrasmall superparamagnetic iron oxide nanoparticles; USPIO-FUCO, USPIOs coated with fucoidan*.

**Figure 4:**
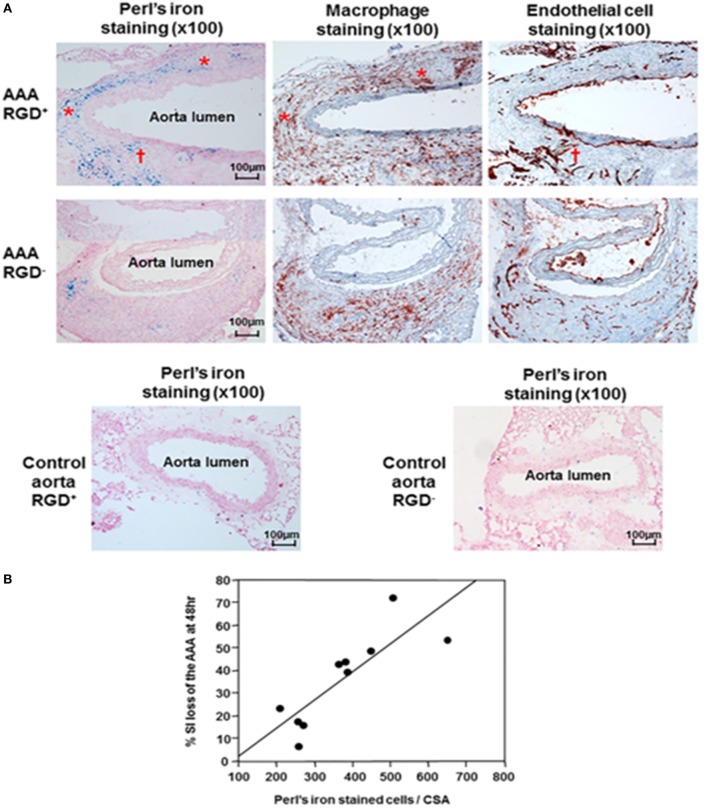
Histological analysis of abdominal aortic aneurysms and correlation with $T_{2}^{*}$ signal loss on MRI. **(A)** Immunohistochemical AAA staining showed mural macrophage infiltration and angiogenesis within the AAA wall. Perl’s iron staining showed greater accumulation of RGD-HFn-Fe_3_O_4_ in the media and adventitia of AAA wall (AAA RGD^+^) compared to HFn-Fe_3_O_4_ (AAA RGD^−^), co-localizing with both macrophages (asterisks) and areas of angiogenesis (dagger). The control aortic wall showed minimal Perl’s iron staining in both RGD^+^ and RGD^−^ groups. **(B)** There was a close correlation between the total number of Perl’s iron-stained cells and % signal intensity loss in the AAA (*n* = 10, *r* = 0.83, *P* = 0.003). Reprinted from Kitagawa et al. (30), under the terms of the Creative Commons Attribution Non-Commercial License (CC BY-NC). Abbreviations: AAA, abdominal aortic aneurysm; MRI, magnetic resonance imaging; RGD-HFn, Arg–Gly–Asp-conjugated human ferritin nanoparticle; *r*, Pearson’s correlation coefficient; $T_{2}^{*}$, transverse relaxation time constant.

Two further studies assessed Arg–Gly–Asp (RGD)-conjugated human ferritin nanoparticle (HFn) enhanced near-infrared fluorescence imaging (65) and MRI imaging (30) of AAAs in the ang II-infused Apo E^−/−^ mouse model. In a proof-of-concept analysis, RGD-HFn was shown to co-localize with infiltrating macrophages and angiogenesis as assessed by immunohistochemistry (30, 65). Mural macrophage infiltration and angiogenesis were assessed by CD-11b and CD-31 immunohistochemistry (Figure 4). In addition, RGD^+^ AAAs exhibited higher iron staining in the media and adventitia compared to the RGD^−^ ones, and this was correlated with the percentage signal intensity loss (30). Nahrendorf et al. employed dextran-coated cross-linked iron oxide nanoparticles (CLIO) labeled with fluorine-18 (^18^F) to image AAAs within the same mouse model (66). They reported that ^18^F-CLIO had a high affinity for inflammatory macrophages and enhanced AAA imaging by positron emission tomography-computed tomography (PET-CT). They reported that PET signal intensity was a good predictor of AAA growth. AAAs with marked uptake of F-CLIO had significant later expansion. They also reported a weak correlation between the AAA diameter measured by CT and the macrophage PET signal. Importantly, their study suggested that ^18^F-CLIO uptake by macrophages was a useful marker of subsequent AAA progression.

Together, these studies suggest that contrast agents employing NPs to target inflammation and thrombosis have potential to visualize pathological processes in AAAs. However, whether these imaging agents can advance current methods of identifying high risk AAAs remains to be established (67).

### Human-Associated Studies

A number of clinical studies have assessed the potential of NPs for enhancing imaging of AAAs (Table 2). Truijers and colleagues conducted a clinical study investigating the uptake of macrophage-specific USPIO in 11 patients with an aneurysm (aortic, *n* = 6, and iliac, *n* = 5) and 11 age-matched non-aneurysmal controls (44). They reported that USPIO-enhanced MRI identified large number of USPIO-positive quadrants within the walls of two AAAs but limited or no USPIO uptake in the other aneurysmal patients and the controls. They hypothesized that the observed variation in USPIO uptake may be due to selective uptake of the NPs within AAAs with a propensity for growth or rupture (44). Sadat et al. reported the USPIO-enhanced MRI of the inflamed aortic wall of 13 patients with AAA. They also reported that quantitative $T_{2}^{*}$ (transverse relaxation time constant) and T2 values (decay of transverse magnetization) provided a reliable quantitative method for assessing USPIO uptake within AAAs (32). These findings provide some evidence that USPIO-enhanced MRI may visualize severe inflammation within AAAs, although no histological confirmation of uptake was available (43). SPIO-enhanced MRI has also been reported to enable successful visualization of AAA ILT morphology and localization of phagocytic leukocytes in a study of 15 patients (68). The authors reported a significant decrease in the MRI contrast-to-noise ratios in both the ILT and the deeper thrombus following SPIO administration, which was positively correlated with the levels of MMP-2, MMP-9, and CD68^+^ macrophages (68).

**Table 2:** **Examples of human studies assessing the use of nanoparticles (NPs) in AAA imaging**.

AAA process	Target	Sample size	NP	Imaging mode	Findings
Inflammation	Macrophages	22^a^	USPIO	MRI	USPIO-enhanced MRI detected inflammation in AAA (44)
Inflammation	Macrophages	13	USPIO	MRI	Significant difference in decay of transverse magnetization pre- and post-USPIO infusion in AAA (43)
Inflammation	Leukocytes	15	SPIO	MRI	SPIO-enhanced MRI detected inflammation in AAA (68)
Inflammation	Macrophages	27	USPIO	MRI	Patients with marked USPIO uptake had threefold higher AAA growth rate (33)
Inflammation	Macrophages	15	USPIO	PET-CT and MRI	F-FDG PET-CT appears to target glycolytic macrophages while USPIO-enhanced MRI appears to target phagocytic macrophages
					Both methods improved AAA imaging (34)

*^a^11 with aneurysm and 11 aged-matched non-aneurysmal controls*.

*AAA, abdominal aortic aneurysm; F-FDG, ^18^F-fludeoxyglucose; MRI, magnetic resonance imaging; PET-CT, positron emission tomography-computed tomography; SPIO, superparamagnetic iron oxide nanoparticles; USPIO, ultrasmall superparamagnetic iron oxide nanoparticles*.

**Figure 5:**
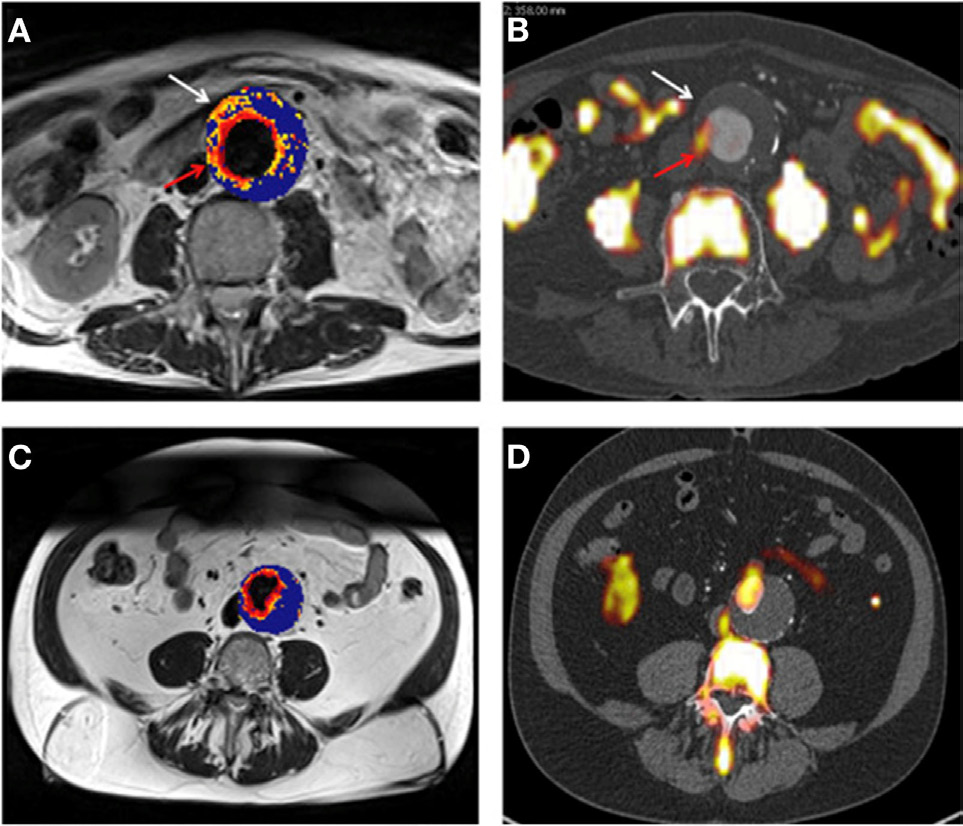
**(A,B)** Representative magnetic resonance imaging (MRI) **(A)** and fused positron emission tomography-computed tomography (PET-CT) **(B)** scans from the same patient with an abdominal aortic aneurysm (AAA). Ultrasmall superparamagnetic particles of iron oxide (USPIO) uptake, defined by percentage change in $T_{2}^{*}$ are demonstrated using a color scale. Changes in $T_{2}^{*}$ value over the threshold (59%) are presented on a graduated (yellow-red) color scale and data below the threshold appears blue. Corresponding 18F-fludeoxyglucose (FDG) activity (red arrow) is shown **(B)**. Differences in the location of regions of uptake between the techniques are apparent, as marked by the white arrow. Panels **(C,D)** are corresponding MRI and fused PET-CT slices from the same patient who had no USPIO or 18F-FDG uptake in the wall of the AAA, with uptake limited to the peri-luminal area. Reprinted from McBride et al. (34), under the terms of the Creative Commons Attribution-Non-Commercial-No Derivatives License (CC BY-NC ND).

In a pilot study involving 27 male patients with AAAs (AAA diameter >4 cm) recruited from a surveillance program, it was reported that USPIO-enhanced MRI identified AAAs that subsequently rapidly expanded (33). The authors reported that patients with significant mural uptake of USPIO had a threefold higher AAA growth rate (measured over 6 months) compared to those with no or non-specific USPIO uptake (33). They also reported that USPIO co-localized with CD68^+^ inflammatory macrophages in the aneurysmal wall of patients who had open AAA repair, which was verified by immunohistochemistry (33). In a more recent study from the same group, the authors compared ^18^F-Fludeoxyglucose (^18^F-FDG) combined PET-CT (^18^F-FDG PET-CT) and USPIO-enhanced MRI for assessing aortic tissue inflammation in 15 patients with asymptomatic AAA [mean AAA diameter 4.6 cm (Figure 5)] (34). Both areas of increased USPIO uptake with and without co-localization with ^18^F-FDG were identified in the same quadrant within the aortic wall. They further reported that both ^18^F-FDG PET-CT and USPIO-enhanced MRI were equally efficient in identifying AAA associated inflammation; however, their data suggested that the different modalities targeted distinct macrophage phenotypes (i.e., those exhibiting glycolytic activity or those exhibiting phagocytic activity, respectively) (34).

Collectively, these studies suggest that NPs can be localized in some AAAs and may have a role in identifying higher risk AAAs. However, the studies currently published are pilot or feasibility studies with small sample sizes. It remains to be shown whether these findings can be validated in large scale clinical trials.

### The Potential of Employing NPs to Identify High Risk AAA

Currently, available methods of imaging AAAs assess anatomical features, such as maximum AAA diameter and volume (67). Most AAAs identified by screening studies are small. There is a dearth of available imaging techniques that can identify pathological features of AAAs. The use of NPs as contrast agents could provide a means to identify such pathological features. Such functional imaging could provide a means to identify AAAs likely to rupture or grow more rapidly which should undergo early elective AAA repair. Larger studies are, however, needed to assess the value of this imaging in a more robust way. We have previously highlighted the difficulties in translating findings from pre-clinical animal models into practice (69), particularly as there is no current ideal AAA animal model. If further clinical studies are encouraging, there are also other challenges to overcome before the widespread clinical application of NP-enhanced imaging. These include cost, large scale production difficulties, and putative systemic toxicity of these agents (70, 71). The design of nanomaterials able to specifically target high risk AAAs (15) is complicated by the multifactorial nature of AAA. Problems with non-specific uptake of NPs by non-target tissues can complicate signal quantification. Hence, image acquisition, quantification, and analysis methods need standardization to ensure rigor, reproducibility, validity, and reliability. Another potential problem is that NP localization is unlikely to reflect the hemodynamic forces on the aortic wall, which are also relevant to AAA rupture risk (72).

Nanoparticle-associated toxicity has been suggested to depend on a number of factors including NP size, composition, or charge (17, 73). Gold NPs, for example, are implicated in the induction of reactive oxygen species and autoimmunity (74). Cationic liposomal NPs can interact with lipoproteins, serum proteins, and ECM, resulting in aggregation and oxidative stress with consequent non-target tissue damage (75, 76). None of these toxic effects were reported in any of the human studies investigating AAA reviewed in this article. Assessment of toxicity is further compounded by the diversity of the constituents used in NPs constructs, since there are infinite numbers of combinations of nanomaterials possible (77). Hence, the probability of negative interactions is quite high (17, 77). However, previous studies suggest that iron oxide NPs are degraded within a week by macrophage lysosomes and the iron incorporated into hemoglobin (78). They are also rapidly cleared from the circulation by the reticular endothelial system albeit in a dose-dependent manner (79, 80). These mechanisms may attenuate any toxicity associated with this form of NP. While NP-associated toxicity is of concern, evidence suggests that iron oxide NPs, such as USPIOs provide application possibilities with marginal potential for toxicity (33, 43, 78–80). In our opinion, additional studies using animal models of AAA and early phase clinical trials should focus on improving and fine tuning iron oxide NPs specifically USPIOs to better assess their efficacy in the functional and molecular imaging of AAA. These studies need to be better designed with adequate sample size. The potential of using these agent as dual imaging and therapeutic drug delivery agents cannot be overlooked. It may also be feasible to employ NPs as delayed imaging or therapeutic agents that are activated by the detection of key element such as inflammatory macrophages or neutrophils within the aneurysm prone aorta.

In summary, NPs enhanced imaging is currently being actively investigated as a way to identify high risk AAAs. Iron oxide NPs have shown some promise in identifying macrophage within AAAs. Further research is needed to optimize this imaging and more robustly examine its value in clinical management.

## Author Contributions

TE conceived, designed, critically appraised the literature, and wrote the article. FA, AS, FS, and TD critically appraised the literature. JG critically appraised and reviewed the article. All authors revised the article for intellectual content; read and approved the final manuscript for submission.

## Conflict of Interest Statement

The authors declare that the research was conducted in the absence of any commercial or financial relationships that could be construed as a potential conflict of interest.
